# Building resilience together: actionable solutions to tackle workforce challenges and the climate crisis’ impact on health systems within the commonwealth

**DOI:** 10.1080/20523211.2025.2501076

**Published:** 2025-05-30

**Authors:** Grace Grange, Amy Hai Yan Chan, David N. Jones, Beth Ward, Helena Rosado, Victoria Rutter

**Affiliations:** a Commonwealth Pharmacists Association, London, UK; b School of Pharmacy, University of Auckland, Auckland, New Zealand; c Commonwealth Organisation for Social Work, Northampton, UK

**Keywords:** Commonwealth, climate change, disaster planning, healthcare systems, workforce resilience, health workers

## Abstract

The emerging threats facing healthcare systems are becoming increasingly challenging to manage, from climate change effects to overburdened healthcare systems. Fundamental changes to the way that health systems develop and work together are needed to build resilience and ensure that they can withstand and continue to deliver effective care, despite emerging threats. These themes were the focus of the 2024 Commonwealth Civil Society Policy Forum, which brought together members of the Commonwealth Health Professions and Partners Alliance. The forum theme was centred on ‘Actionable Solutions to Building Resilience in Healthcare Systems within the Commonwealth, with an Emphasis on Small and Vulnerable States’. Given the broad network of governmental, non-governmental and civil society organisations across all continents within the Commonwealth, this platform is ideally placed to tackle these global challenges. The purpose of the forum was to develop and agree on key recommendations to take forward to the 2024 Commonwealth Health Ministers Meeting to advocate for policy change. Speakers emphasised the Civil Society’s role in strengthening health systems and urged policymakers to consider, in a global context, the highest priority issues. Six recommendations were agreed upon:

- Addressing the effects of climate change on health.
- Strengthening Health Emergency Capacities.
- Leveraging workforce capability to support health across the whole-life course.
- Bringing younger and older generations together.
- Addressing sustainable availability and employment of healthcare workers.
- Developing a resilient health workforce.
  Civil Society must act as a catalyst for change to build and strengthen healthcare resilience by working collaboratively through innovation, community engagement, promoting equality and diversity and sharing best practice. These six recommendations are a call to action for all civil societies. Through collective action, these principles can be adopted to strengthen every area of healthcare systems to ensure health security and efficiency for our populations.

The emerging threats facing healthcare systems are becoming increasingly challenging to manage, from climate change to overburdened health systems. These issues disproportionately affect small and vulnerable states, exacerbating inequalities (The Commonwealth, 2024). There is an urgent need to act, innovate and transform healthcare systems and workforces to build resilience so they can operate effectively to meet rapidly changing population needs.

Each year, the Commonwealth Health Professions and Partners Alliance (CHPA) brings together members at the Commonwealth Civil Society Policy Forum (CCSPF). The forum is an important opportunity to advance and influence health and wellbeing policy on behalf of the practitioners and citizens that CHPA represents. This forum also provides an opportunity to reinforce the urgent need for continued commitment to decolonising global health, at the systems, organisational and individual levels (Abimbola et al., 2021 and Büyüm et al., 2020). Addressing historical and political imbalances of power and affirming ownership of global health are critical. This ethos underpins the CCSPF’s priorities and shapes its recommendations.

The 2024 forum theme was ‘Actionable Solutions to Building Resilience in Healthcare Systems within the Commonwealth, with an Emphasis on Small and Vulnerable States’. The objectives were to raise awareness of the impact of climate change and other natural disasters on health within Civil Society, highlight current health workforce challenges across the Commonwealth and provide an opportunity to agree actionable recommendations to present at the Commonwealth Health Ministers Meeting (CHMM). This commentary discusses the six agreed recommendations presented to Commonwealth Health Ministers.

## 1. Recommendation 1 – addressing the effects of climate change on health

Globally, we are increasingly seeing the negative consequences of climate change on health. Disasters such as rising sea levels, extreme weather and poor air quality are having damaging effects on healthcare infrastructure, and the physical and mental health of communities, with a disproportionate effect on vulnerable populations (Seervai et al., 2022). The impact of climate change must be prioritised in the development of all policies and resources if its damaging consequences are to be slowed down. Adaptation and mitigation strategies must be put in place to build more resilient communities as well as increased investment in education and developing robust healthcare facilities, particularly in small island states.

## 2. Recommendation 2 – strengthening health emergency capacities

Climate change significantly impacts the provision of medicines and supply chains, disrupting the safe and effective delivery of healthcare. Examples include global warming enabling resistant pathogens to replicate more rapidly, exacerbating antimicrobial resistance, and extreme weather events preventing routine healthcare delivery. CHPA calls for Commonwealth states to develop preparedness plans to strengthen the resilience of supply chains and maintain access to services in disaster scenarios to enable a sustainable response. The World Health Organization (WHO) benchmarks for Strengthening Health Emergency Capacities and Commonwealth Heads of Procurement Network are central to the development of these plans (WHO, 2023).

## 3. Recommendation 3 – leveraging workforce capability to support health across the whole-life course

We are living in a globally aging population with an increasing burden from non-communicable diseases placing higher demand on healthcare systems. To support communities to maintain their health, there must be a greater focus on the prevention, early detection, and effective management of disease. CHPA calls for national action plans that include innovation and maximising the capabilities of the entire workforce. Key to this is effective collaboration between health and other sectors, leveraging the strengths of different health workers and empowering communities to build resilience.

## 4. Recommendation 4 – bringing younger and older generations together

Opportunities for intergenerational interaction and learning should be prioritised to create healthier communities. The benefits of intergenerational integration include shared learning, which encourages both young and older people to establish positive emotions, attitudes and values. Evidence suggests that intergenerational practice and shared learning can reduce social exclusion and loneliness (Abrahams et al., 2007) as well as create safe environments for exchanging life skills, as demonstrated by the Strive Befriending Service Intergenerational Project (NHS Health Scotland, 2025). This is key to developing robustness in Commonwealth countries by enabling families, health workers and the communities they serve to support each other at all stages of the life course.

## 5. Recommendation 5 – addressing sustainable availability and employment of healthcare workers

The health workforce crisis is a real and growing challenge. Evidence shows that there is an increase in workforce attrition due to aging, burnout, poor mental health and global migration (Hambleton et al., 2023; WHO, 2024a). There are increasing pressures on healthcare workers including: escalating and increasingly complex clinical presentations and patient demands; heavy workloads; staff shortages and poor support systems. These challenges place high demands on staff, affecting mental wellbeing and resilience, which has been further exacerbated by the COVID-19 pandemic (Baskin & Bartlett, 2021; Lu et al., 2020). Insufficient funding and recruitment paired with escalating demands on services further contributes to the pressures on already stretched healthcare systems. Ethical recruitment practices are key and the CHPA recommends working collaboratively with the Commonwealth network and civil society to develop policies and global partnerships to share learning, upskill and increase workforce retention. There must be an emphasis on ensuring fairness and mutual benefit when recruiting from low- and middle-income countries, and guidelines need to be developed, in line with the WHO Global Code of Practice on the International Recruitment of Health Personnel (WHO, 2010), to ensure ethical recruitment occurs.

The WHO Director-General’s remarks from CHMM in May 2024 highlighted the long-standing gaps between the demand for and supply of health and care workers in most countries, calling on the need for investment, particularly in education and working conditions (WHO, 2024b). Several CHPA members welcomed these commitments to the health and social care workforce (Commonwealth Pharmacists Association, 2024 and Commonwealth Organization for Social Work, 2024).

## 6. Recommendation 6 – developing a resilient health workforce

Evidence shows that up to 62% of health and social workers experience some form of abuse, both physical and non-physical, throughout their career (Liu et al., 2019). This violence against health workers is increasing, with damaging effects on workers’ mental and physical wellbeing and therefore a negative impact on healthcare system resilience. Further problems stem from insufficient workforce capacity, with small island states disproportionately impacted. New strategies are required to attract people into health professions. This final recommendation from the CHPA forum emphasises the need to establish multi-disciplinary national and local evidence-based interventions and policies to ensure adequately resourced, safe and equitable work environments that promote health and wellbeing. These should include creating inclusive jobs and recruitment policies, and addressing pay gaps and poor working conditions.

The CHMM pledged joint commitments to build resilient healthcare systems in small and vulnerable states, which included the health and social care workforce. More specifically, this included to ‘prioritise the wellbeing of health and care workers and strive for gender equality while ensuring access to safe and decent working conditions, career pathways, work-life balance, and protected labour rights including parental leave’ (The Commonwealth, 2024).

## Conclusions

Six key recommendations were agreed upon at the CCSPF in March 2024, which emphasise the need for robust actions to address the impact of climate change and natural disasters, workforce challenges and healthcare system resilience throughout the Commonwealth. Key areas of focus for governments recommended in the CHPA submission and reflected in the communiqué included: (1) planetary health and climate change (paras 24–28, 61); (2) global health security and emergency responses post-pandemic (paras 1, 3, 67); (3) supporting health and wellbeing across the whole life course, including physical and mental health and non-communicable diseases (paras 11, 14, 18–23, 34); (4) bringing younger and older generations together and developing a roadmap on ageing well (para 58) and (5&6) sustainability and resilience of the healthcare workforce including ethical recruitment, migration and equality and inclusion (paras 13, 37–41, 56). The forum’s recommendations reflected civil society’s priorities and influenced the outcome of the 2024 Commonwealth Health Ministers meeting to support actions needed to build healthcare resilience across the Commonwealth, ahead of the 2024 World Health Assembly.

## Data Availability

The datasets used and/or analysed during the current study are available from the corresponding author on reasonable request.
